# Supplementary figures and images for: Dual engagement of the nucleosomal acidic patches is essential for deposition of histone H2A.Z by SWR1C
Source: eLife. 2024 May 29;13:RP94869. doi: 10.7554/eLife.94869 (PMC11139478; doi:10.7554/eLife.94869)

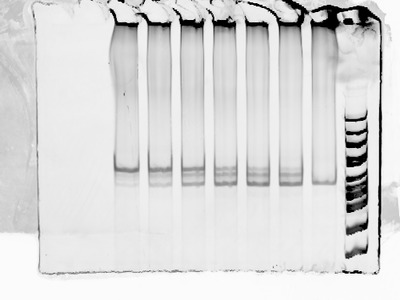

Supplement: Figure 1—figure supplement 1—source data 2. [file elife-94869-fig1-figsupp1-data2.zip › Figure 1-Figure Supplement 1-Source Data 2/Figure 1-Figure Supplement 1-Panel A raw.jpg]

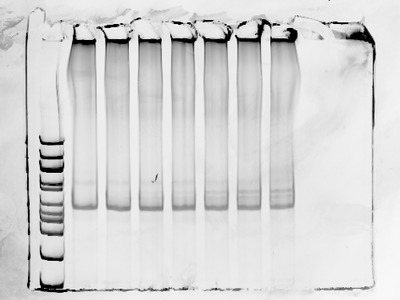

Supplement: Figure 1—figure supplement 1—source data 2. [file elife-94869-fig1-figsupp1-data2.zip › Figure 1-Figure Supplement 1-Source Data 2/Figure 1-Figure Supplement 1-Panel C raw.jpg]

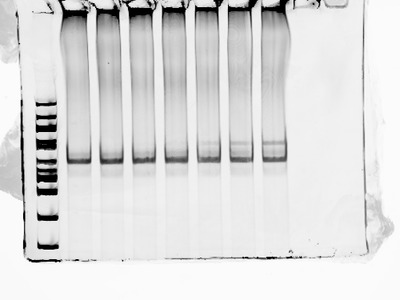

Supplement: Figure 1—figure supplement 1—source data 2. [file elife-94869-fig1-figsupp1-data2.zip › Figure 1-Figure Supplement 1-Source Data 2/Figure 1-Figure Supplement 1-Panel B raw.jpg]

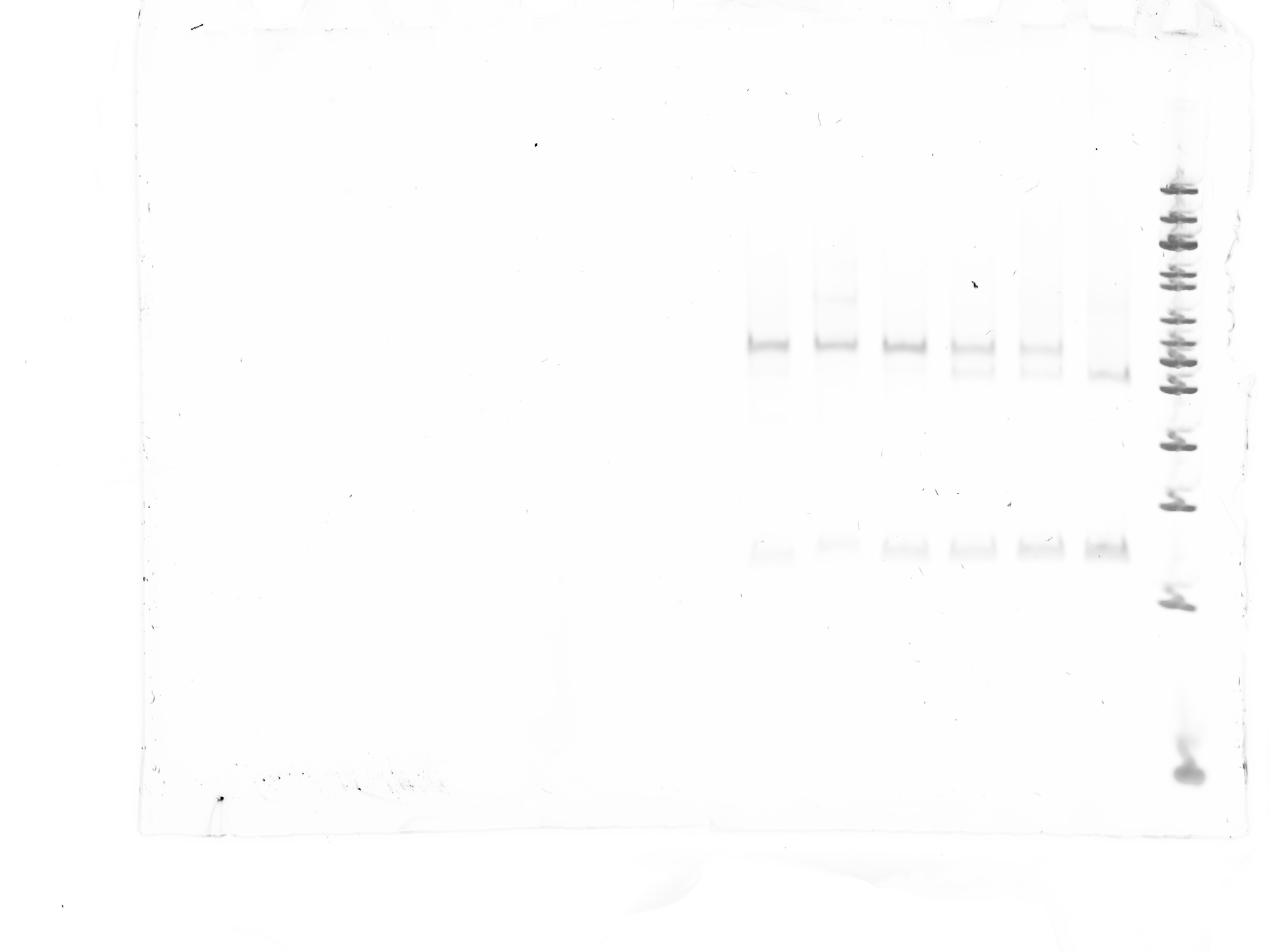

Supplement: Figure 1—figure supplement 2—source data 1. [file elife-94869-fig1-figsupp2-data1.zip › Figure 1-Figure Supplement 2-Source Data 1/Figure 1-Figure Supplement 2-Panel C raw.tif]

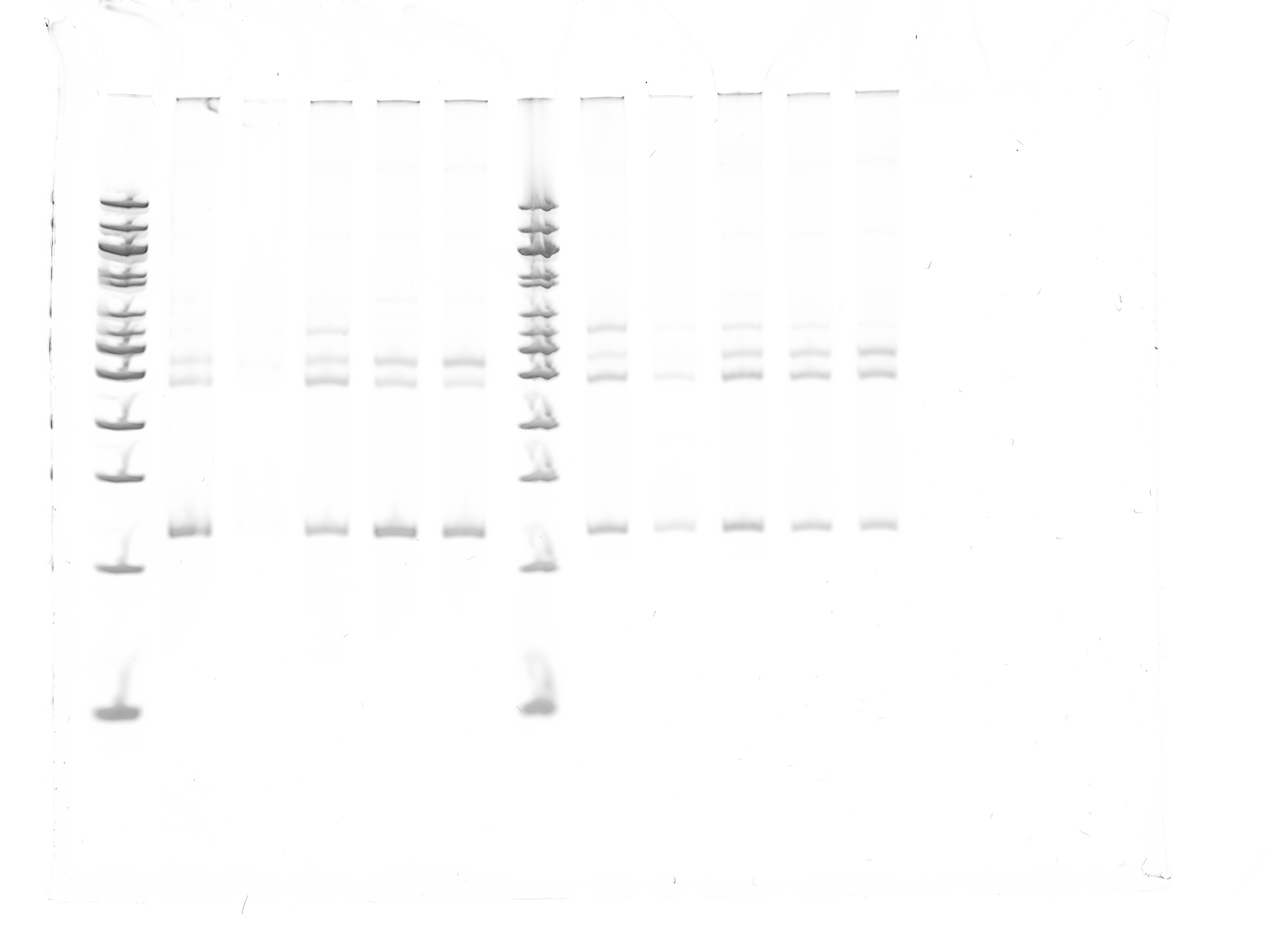

Supplement: Figure 1—figure supplement 2—source data 1. [file elife-94869-fig1-figsupp2-data1.zip › Figure 1-Figure Supplement 2-Source Data 1/Figure 1-Figure Supplement 2-Panel B raw.tif]

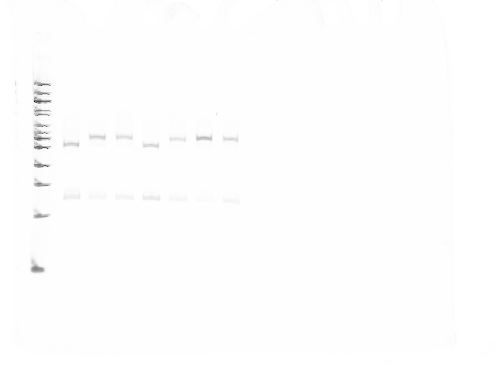

Supplement: Figure 1—figure supplement 2—source data 1. [file elife-94869-fig1-figsupp2-data1.zip › Figure 1-Figure Supplement 2-Source Data 1/Figure 1-Figure Supplement 2-Panel D raw.tif]

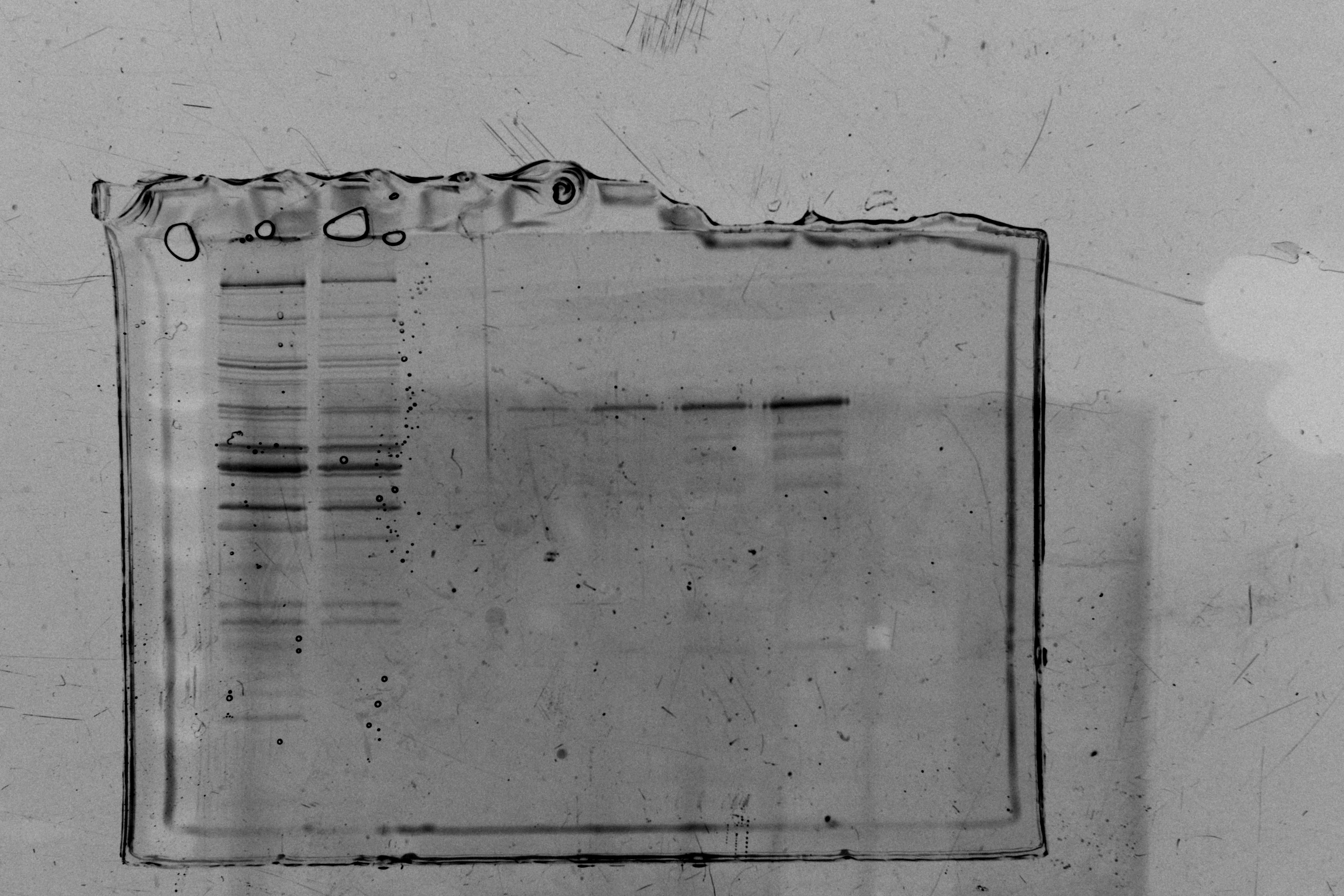

Supplement: Figure 3—figure supplement 1—source data 1. [file elife-94869-fig3-figsupp1-data1.zip › Figure 3-Figure Supplement 1-Source Data 1/Figure 3-Figure Supplement 1-Gel 4 raw.tif]

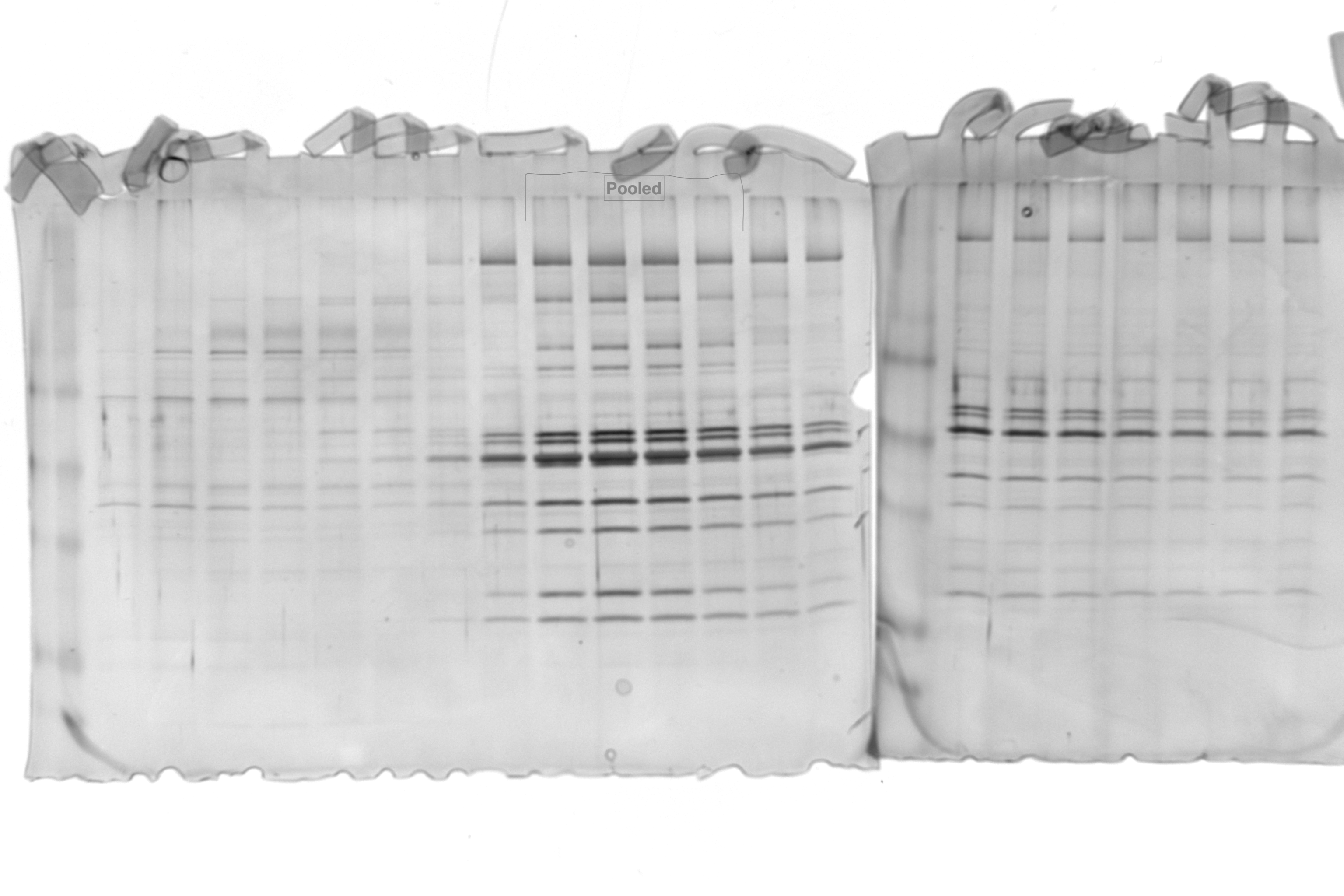

Supplement: Figure 3—figure supplement 1—source data 1. [file elife-94869-fig3-figsupp1-data1.zip › Figure 3-Figure Supplement 1-Source Data 1/Figure 3-Figure Supplement 1-Gel 1 raw.tif]

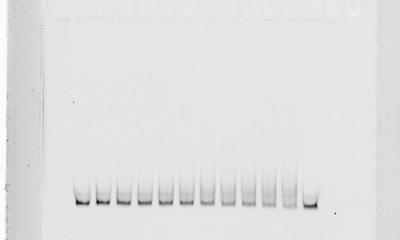

Supplement: Figure 4—source data 2. [file elife-94869-fig4-data2.zip › Figure 4-Source Data 2/Figure 4-Panel D raw.jpg]

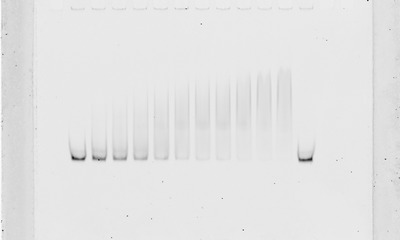

Supplement: Figure 4—source data 2. [file elife-94869-fig4-data2.zip › Figure 4-Source Data 2/Figure 4-Panel B raw.jpg]

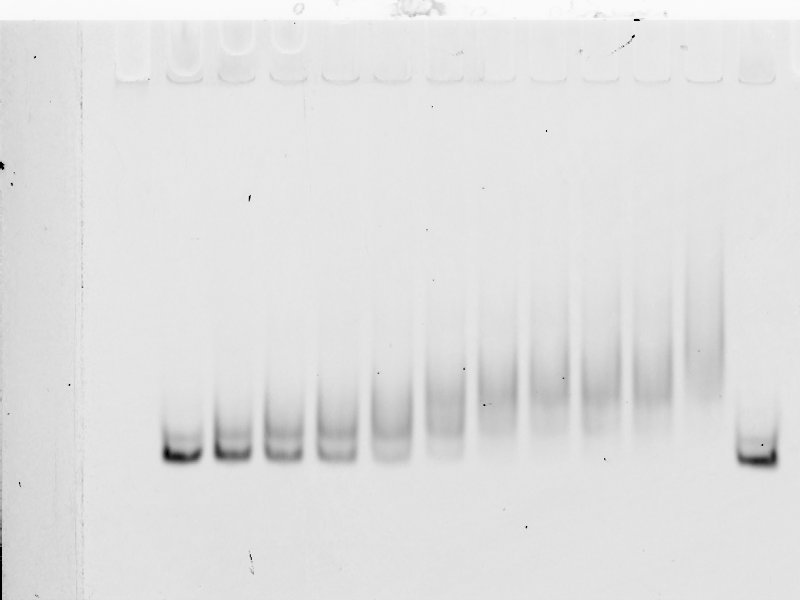

Supplement: Figure 4—source data 2. [file elife-94869-fig4-data2.zip › Figure 4-Source Data 2/Figure 4-Panel A raw.tif]

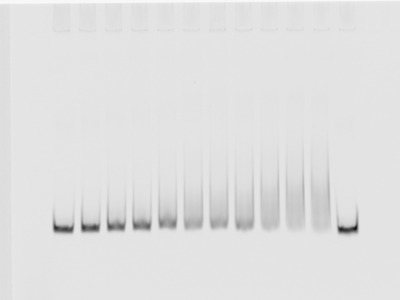

Supplement: Figure 4—source data 2. [file elife-94869-fig4-data2.zip › Figure 4-Source Data 2/Figure 4-Panel C raw.jpg]

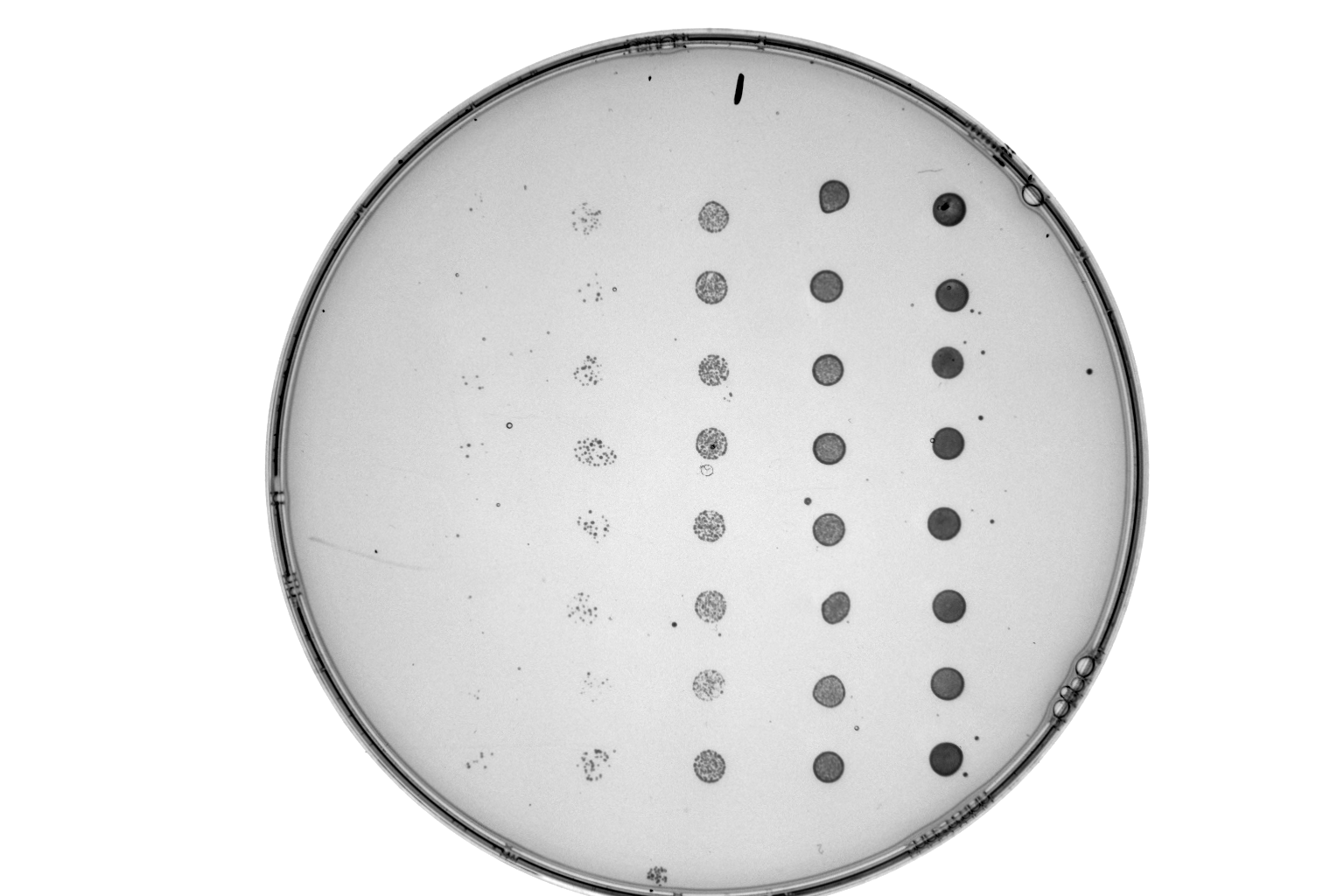

Supplement: Figure 8—source data 1. [file elife-94869-fig8-data1.zip › Figure 8-Source Data 1/Figure 8-Left Panel raw.tif]

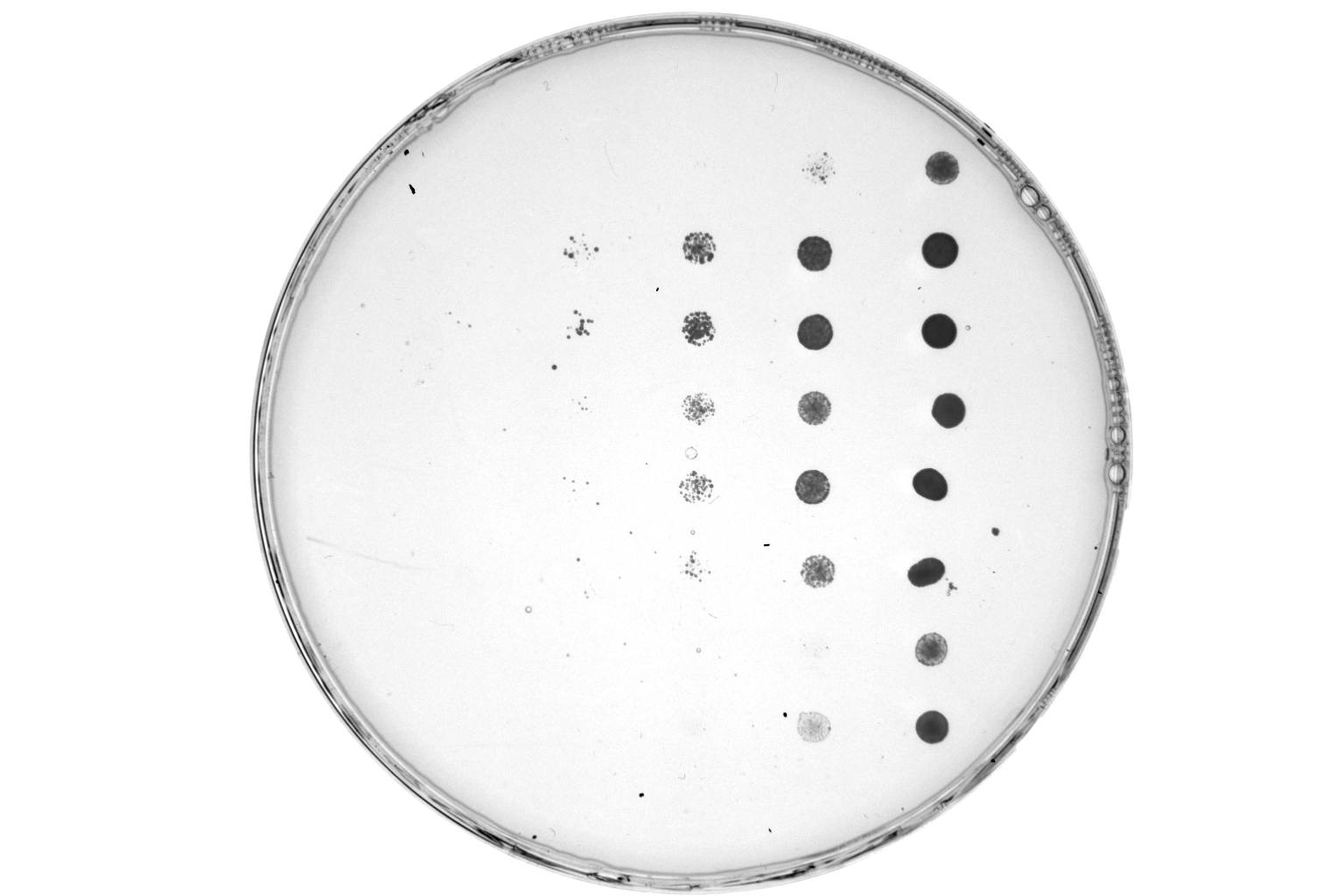

Supplement: Figure 8—source data 1. [file elife-94869-fig8-data1.zip › Figure 8-Source Data 1/Figure 8-Right Panel raw.tif]
